# Chitosan-Graphene Oxide Dip-Coated Polyacrylonitrile-Ethylenediamine Electrospun Nanofiber Membrane for Removal of the Dye Stuffs Methylene Blue and Congo Red

**DOI:** 10.3390/nano13030498

**Published:** 2023-01-26

**Authors:** Maadri A. Pathirana, Nethmi S. L. Dissanayake, Nandula D. Wanasekara, Boris Mahltig, Gayani K. Nandasiri

**Affiliations:** 1 Department of Textile and Apparel Engineering, University of Moratuwa, Moratuwa 10400, Sri Lanka; 2 Faculty of Textile and Clothing Technology, Hochschule Niederrhein—University of Applied Sciences, 47707 Krefeld, Germany

**Keywords:** polyacrylonitrile, electrospinning, methylene blue, Congo red, graphene oxide

## Abstract

Textile wastewater accommodates many toxic organic contaminants that could potentially threaten the ecosystem if left untreated. Methylene blue is a toxic, non-biodegradable, cationic dye that is reportedly observed in significant amounts in the textile effluent stream as it is widely used to dye silk and cotton fabrics. Congo red is a carcinogenic anionic dye commonly used in the textile industry. This study reports an investigation of methylene blue and Congo red removal using a chitosan-graphene oxide dip-coated electrospun nanofiber membrane. The fabricated nanocomposite was characterized using Scanning Electron Microscopy (SEM), FT-IR Spectroscopy, Raman Spectroscopy, UV-vis Spectroscopy, Drop Shape Analyzer, and X-ray Diffraction. The isotherm modeling confirmed a maximum adsorptive capacity of 201 mg/g for methylene blue and 152 mg/g for Congo red, which were well fitted with a Langmuir isotherm model indicating homogenous monolayer adsorption.

## 1. Introduction

Contaminated wastewater poses a great threat to the ecosystem and human health. Organic dye contaminants present in effluent streams connected to the textile, paper, printing, cosmetic, and food industries contain particles that are high in resistance to oxidizing agents, photodegradation, heat, and biodegradation, which in turn create potential health risks to humans. The annual production of commercial dyes amounts to over 7 × 10^5^ tons of which the textile industry accounts for two-thirds of the consumption [1]. It has also been estimated that approximately 10–20% of the manufactured dye is released yearly into effluent streams [2]. In the literature, diverse and varied chemical, physical, and biological dye treatment techniques have been reported, i.e., chemical oxidation [3], coagulation [4], photodegradation [5], biodegradation [6], adsorption [7,8,9], and membrane separation among which physical adsorption methods are widely used owing to their cost-effectiveness, simplicity, and environmental resistance. Moreover, as dye molecules are highly resistant to oxidizing agents and biodegradation, chemical and biological water treatment methods are not considered pragmatic solutions.

**Figure 1:**
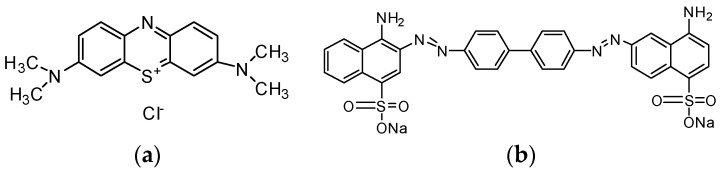
Chemical Structure of (**a**) methylene blue and (**b**) congo red.

The literature on adsorbents targeted for removing either anionic or cationic dyes is available in abundance. However, there is limited research on simultaneous anionic and cationic dye removal techniques. Methylene blue (Figure 1a) is a toxic, non-biodegradable, cationic dye that is reportedly found in significant amounts in the textile effluent streams as it is widely used to dye cotton and silk fabrics [10]. Previous studies reported significant health issues, viz. nausea, jaundice, profuse sweating, burning sensations, quadriplegia, cyanosis, and methemoglobinemia, linked with methylene blue exposure [11,12,13]. Congo red (Figure 1b) is an azo-based anionic dye commonly used in the textile industry [14]. Azo dyes are defined as organic molecules with one or more azo (–N=N–) groups; this category accounts for approximately 60–70% of the total dye consumption [1]. Azo dyes are considered environmental threats as the reductive cleavage of N=N bonds results in the formation of aromatic amines which are toxic and carcinogenic to humans [15]. The reductive cleavage of Congo red, which is classified as the most common azo dye, produces benzidine, which is a known human mutagen and carcinogen [16]. Exposure to Congo red dye is also associated with respiratory problems and skin and eye irritation [17]. Therefore, significant attention should be devoted to facilitating the removal of Congo red and methylene blue from the effluent streams.

With the advancements in nanotechnology, significant research attention has been focused on the usage of nanomaterials as adsorbents to facilitate the removal of organic contaminants. Electrospun polymeric nanofibers (EPNs) are increasingly used in the field of wastewater treatment owing to the unique attributable characteristics of nanofibrous materials, i.e., high theoretical surface area, high porosity, and controllable pore sizes [18]. Electrospinning is a facile and popular technique adopted for the fabrication of EPNs of diameters ranging from 50 nm to 1 μm [19]. In electrospinning, an electric field is used to overcome the surface tension of a charged polymeric solution and elongate the jet into thin nanofibers toward a grounded collector for which the feed flow rate, tip-to-collector distance, polymer concentration, and applied voltage can be controlled to achieve the desired morphology. Research and technological progress on the upscaling capacity of the electrospinning process has significantly enhanced its potential as a nano adsorbent [20].

Polyacrylonitrile (PAN) is a synthetic polymer widely used for fabricating EPNs owing to its excellent thermal and mechanical properties [21,22]. The capacity of electrospun PAN nanofibers to remove dye contaminants from effluent streams is restricted by its low surface wettability or the lack of available adsorption sites [21]. Surface modification has been proposed as an effective solution to overcome the limitations in its capacity to remove organic contaminants, in which the hydrophilicity of PAN could be enhanced without altering its morphology [23,24,25]. As reported in the previous literature, the adsorbent capacity of polymeric adsorbents can be significantly enhanced by introducing oxygen- or nitrogen-containing functional groups among which amino-functionalization is highly favored for its capacity of forming strong complexes with organic contaminants [25,26,27]. Amino-functionalized PAN electrospun nanofibers were fabricated for the first time by Almasian et al. [25] to facilitate the removal of anionic dyes. It was demonstrated that surface functionalization plays a pivotal role in enhancing the adsorption capacity of PAN EPNs. An amino-functionalized porous PAN nanofiber membrane was fabricated by Mahmoodi et al. [28] through the inclusion of sodium carbonate, which also displayed a superior adsorption capacity for anionic dyes. In a study conducted by Patel and Hota [29], it was shown that the adsorption capacity of modified PAN nanofibers has a direct correlation with the density of amino groups in which PAN functionalized through ethylenediamine (EDA) reported the highest adsorptive capacity for Congo red dye, which amounted to 130 mg/g [29].

**Figure 2:**
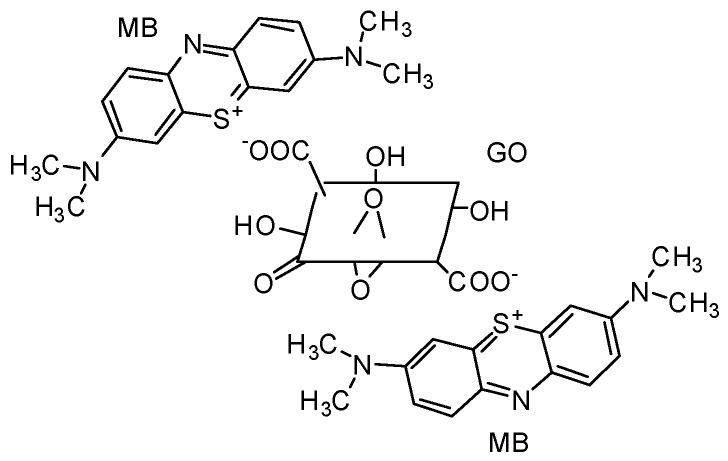
Electrostatic interaction between graphene oxide and methylene blue.

A significant number of adsorbents for methylene blue are reported in the literature, such as chitosan [30], zeolite [31], and carbon-based adsorbents, i.e., carbon nanotubes [32], activated carbon [33,34], biochar [35], and graphene oxide [36]. In comparison to other materials, carbon-based adsorbents are more extensively used for organic dye removal owing to their easy accessibility, facile synthesis, and inherent porosity [37]. Among the reported carbon-based adsorbents for methylene blue, graphene oxide is emphasized in the literature for its high surface area, two-dimensional structure, and oxygen-containing functional groups that can form strong electrostatic interactions with cationic dyes (Figure 2).

**Figure 3:**
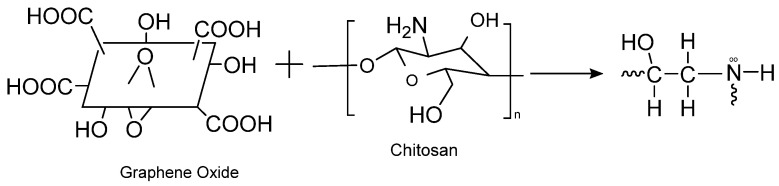
Crosslinking mechanism between chitosan and graphene oxide.

The recovery process of GO from the effluent is challenging if used in its raw form as it tends to form stable colloids that inhibit phase separation. As per the claims of the previous literature studies, adsorbents fabricated through cross-linking GO with different polymers could serve as an attractive solution to overcome limitations in the usage of GO as an adsorbent [38]. Chitosan (Ch) is a biopolymer decorated with amino groups possessing promising applications in wastewater treatment. As reported by Shao et al. [39], the epoxy groups on GO can crosslink with Ch through its amino groups (Figure 3). The adsorptive potential of GO encapsulated in a chitosan matrix toward methylene blue was reported in several research studies [40,41,42].

As this research aims to deliver a structure with optimized adsorptive capacities for methylene blue and Congo red, a composite membrane incorporating both Ch-GO and functionalized PAN should be fabricated. Several studies reveal that chitosan-related composites could strongly adhere to PAN through hydrogen bonds [30,43,44]. The literature proposes dip coating as a facile, energy-intensive, and economical coating process to fabricate thin nanoparticle-coated substrates [45,46,47,48]. The properties of such composite polymer membranes, i.e., mechanical strength, adsorption capacity, and wettability are stated to be dependent on the choice in raw materials, modifications, and fabrication methods [49,50].

The present study explores the possibility of using a novel two-layered hybrid nanocomposite membrane consisting of a dip-coated graphene-chitosan (GO-Ch) top coating and a PAN-EDA electrospun bottom layer to facilitate the simultaneous removal of methylene blue and Congo red dyes. The novelty of the research can be attributed to the unique engineered structure of the proposed solution, i.e., the two-tiered adsorbent design with each layer having chemical and structural properties to optimize the adsorption of methylene blue or Congo red. Moreover, the proposed structure targets the removal of both cationic dyes and anionic dyes, which is an application with limited available examples in the literature. An electrospun nanofiber membrane is strategically selected as the bottom layer due to its high surface area, controllable pore structures, regeneration capacity, and due to its inherent substantivity for organic dyes [18]. As the top layer consists of a chitosan-based composite, it can tightly adhere to the PAN electrospun surface through dipole interactions [36,37,38]. The study further expects to investigate the effect of pH, temperature, and contact time of the nanocomposite membrane on methylene blue and Congo red removal and determine the maximum adsorption capacity of the proposed solution through isotherm modeling.

## 2. Materials and Methods

### 2.1. Materials

Polyacrylonitrile powder (Mw = 150,000) was purchased from Shandong Natural Micron Pharm Tech Co., LTD, China. The chitosan was synthesized using shrimp shells retrieved from domestic waste. The graphene oxide was synthesized with graphite powder purchased from Neochem International (Pvt) Ltd., Battaramulla, Sri Lanka. Hydrochloric acid (HCl, 37%), Sodium Hydroxide Pellets (NaOH, 97%), Dimethylformamide (DMF, 99.8%), Ethylenediamine (EDA, 99%), Acetic acid (Glacial, 100%), Phosphoric acid (H_3_PO_4_, 85%), Sulfuric acid (H_2_SO_4_, 99%), Potassium permanganate (KMnO_4_, 99.9%), Hydrogen peroxide (H_2_O_2_, 30%), and Ethanol (C_2_H_5_OH, 99%) were purchased from Sigma Aldrich, United States. All the chemicals were used without any further purification.

### 2.2. Extraction of Chitosan

The collected shrimp waste from Pitipana, Sri Lanka was thoroughly washed using tap water and ground using a mortar and pestle. The crushed shrimp shells were kept in polyethylene bags at room temperature (26 °C) for a duration of 24 h to promote partial autolysis, which is a preparatory step for extracting chitosan to improve the quality of the yield [51]. The extraction procedure was carried out in three consecutive steps, i.e., demineralization, deproteinization, and deacetylation, in accordance with an established chitosan procedure reported by Hossain et al. [52].

#### 2.2.1. Demineralization

At room temperature, dried shrimp shell powder was treated with 1M HCl (pH 1–3) in a 1:16 (*w/v*) ratio for 24 h. The sample was washed with distilled water until it reached a pH of 6.5–8. The demineralized sample was weighed using an analytical balance after being dried in a vacuum oven at 80 °C.

#### 2.2.2. Deproteinization

The demineralized product was then treated for 48 h at room temperature with 2 M NaOH (pH 11–13) in a 1:14 (*w/v*) ratio. The sample was washed with distilled water until it reached a pH of 6.5–8. The demineralized sample (Chitin) was weighed using an analytical balance after being dried in a vacuum oven at 80 °C.

#### 2.2.3. Deacetylation

The Chitin sample was then treated with 48% NaOH (*w/v*%) (pH 12–14) at room temperature for 48 h with stirring in a 1:16 (*w/v*) ratio. To neutralize the deacetylated sample, it was initially filtered, recovered, and then rinsed with distilled water. Chitosan was obtained by drying the solid sample produced from Chitin in a vacuum oven.

### 2.3. Synthesis of GO

A modified Hummer’s method was used to synthesize graphene oxide from natural graphite powder. The graphite flakes were oxidized for 4 h according to the synthesis methodology reported by Tissera et al. [53]. A magnetic stirrer was used to combine concentrated H_2_SO_4_:H_3_PO_4_ solution in a 9:1 ratio with a graphite flake (1% wt) and KMnO_4_ solution (6% wt). The combination achieves a slight exothermic temperature increment of 35–40 °C. The mixture was then heated to 50 °C and stirred for 4 h. The hue of the solution changed from dark purplish green to dark brown. It was cooled to room temperature by pouring over an ice solution with H_2_O_2_ (30%) to stop the oxidation reaction. The color of the mixture changed to a vivid yellow at this moment, suggesting considerable oxidation of graphite [54]. It was first washed with distilled water, then with HCl (30%), and with ethanol twice after the leftover solid material settled. Before the supernatant was decanted, the mixture was centrifuged for one hour at 9000× *g* rpm. The washing process was repeated until the pH level reached 4–5.

### 2.4. Synthesis of Graphene Oxide-Chitosan Composite Solution

**Figure 4:**
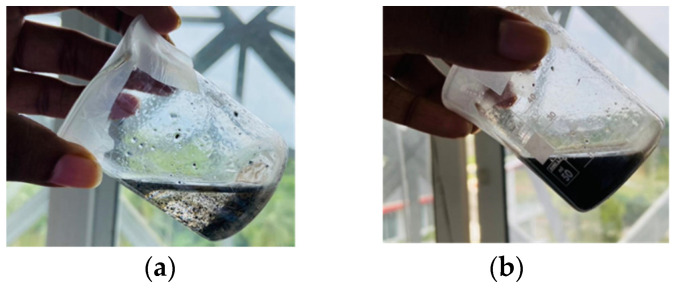
Ch-GO solution (**a**) before and (**b**) after sonication.

The synthesized chitosan powder (Ch) was dissolved in a 4% acetic acid solution at 60 °C for 12 h with continuous stirring to produce a 2% chitosan solution. The synthesized graphene oxide was dispersed in a 1% acetic acid solution to produce a 0.5% GO solution. The produced Ch solution was mixed with the GO solution and the mixture was stirred for 3 h at 600 rpm, then, using a bath sonicator, it was subjected to ultrasound for 4 h to produce a homogenous solution (Figure 4).

### 2.5. Fabrication of Polyacrylonitrile Electrospun Membrane

**Figure 5:**
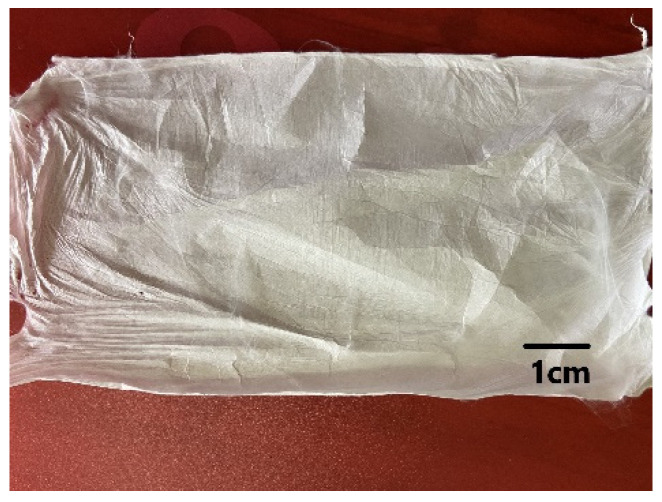
PAN electrospun nanofiber mat.

The PAN polymer solution (10 wt%) was prepared by dissolving PAN polymer powder in Dimethylformamide (DMF) solvent. The PAN solution was stirred for 12 h using a magnetic stirrer to achieve a homogenous polymer solution. A 10 mL syringe with a metallic needle was loaded with the prepared PAN solution. The syringe was attached to a syringe pump (KDS100, Cole Palmer, Vernon Hills, IL, USA) and the feeding rate was fixed at 10 μL/min. A high-voltage power supply (HCP35-35000, FuG Elektronik, Schechen, Germany) was used to apply a 10 kV voltage to the positive terminal needle tip. A collector wrapped in aluminum foil was connected to the negative terminal. The needle tip’s distance from the grounded metal collector was fixed at 15 cm. Ultrafine PAN polymer fibers were generated when high voltage was applied to polymer droplets at the end of the needle tip, followed by solvent evaporation and the collection of nanoscale fibers on the collector (Figure 5).

### 2.6. Surface Functionalization of Electrospun PAN Membrane

**Figure 6:**
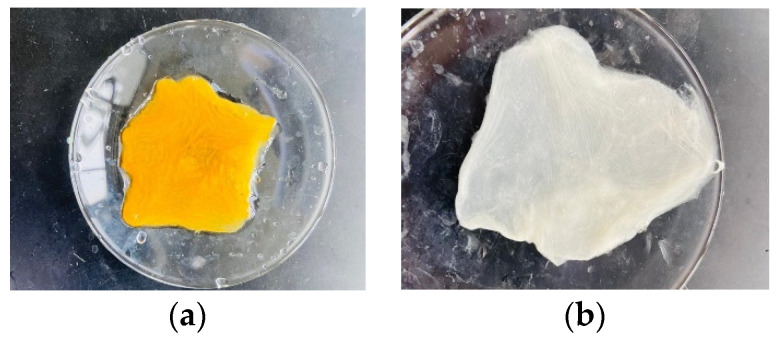
(**a**) NaOH-treated PAN electrospun nanofiber membrane (**b**) EDA-functionalized PAN electrospun nanofiber membrane.

A PAN electrospun mat of size 6 cm × 5 cm was carefully removed from the aluminum foil and submerged in 15% NaOH (50 mL) solution for one hour at 60 °C [55]. The color of the immersed PAN membrane sheet shifted from white to bright yellow (Figure 6a). The treated fiber mat was then rinsed with distilled water and immersed in a 1 M HCl (50 mL) solution for 30 min at room temperature to neutralize the excessive base. The color of the yellowish PAN membrane that had been hydrolyzed was changed to white again. The observations were consistent with the earlier study reported by Patel and Hota [56]. The hydrolyzed PAN nanofiber membrane was then treated for 4–5 h at room temperature with 50 mL of a 10% EDA solution (50 mL) to replace the nitrile groups with the amine groups on the surface of the nanofibers. The PAN electrospun mat was then rinsed in distilled water placed on an aluminum foil and allowed to dry at room temperature (Figure 6b).

### 2.7. Preparation of the Nanocomposite Membrane

The PAN-EDA functionalized membrane of size 6 cm × 5 cm was weighed using a microbalance. The weight of the membrane was measured to be 2 mg. Then, the membrane was secured with a custom-made clamp and was dipped in the prepared Ch-GO solution for 30 s. The coated membrane was then dried in an oven preheated to 60 °C for 30 min and the weight of the dip-coated membrane was measured to be 2.8 mg.

### 2.8. Characterizations

#### 2.8.1. Scanning Electron Microscopy (SEM)

The surface morphology and topography of PAN, PAN-EDA, and Ch-GO dip-coated PAN-EDA membranes were studied using a Scanning Electron Microscope (Carl Zeiss Evo 18 Research SEM, Carl Zeiss Microscopy, White Plains, NY, USA). The SEM images of the samples were taken under an accelerating voltage of 10 kV.

#### 2.8.2. FTIR Spectroscopy

To determine the chemical structure and functional groups of the samples, FTIR spectra were measured using an FTIR Spectrometer (FTIR Bruker Vertex 80, Bruker Corporation, Billerica, MA, USA) in the range from 4000 to 400 cm^−1^.

#### 2.8.3. UV-Vis Spectroscopy

Absorbency studies of methylene blue and Congo red dyes onto Ch-GO dip-coated PAN-EDA composite membrane were conducted using a UV-Visible NIR Spectrometer (UV-3600, Shimadzu, Kyoto, Japan).

#### 2.8.4. X-ray Diffraction

An X-ray diffractometer (D8 Focus X-ray diffractometer, Bruker Corporation, USA) was used to obtain the X-ray diffraction (XRD) patterns of Graphene Oxide. For the analysis, the powdered samples were arranged in a horizontal position and mounted. A scanning speed of 3°/min over a 2θ range of 5–60° was used for the scanning of the diffraction patterns.

#### 2.8.5. RAMAN Spectroscopy

Raman Spectroscopy (BRUKER SENTERRA II -Confocal Raman Microscope, Bruker Corporation, USA) was used to obtain the Raman spectra for the synthesized GO. The spectra were obtained between 500 and 3000 cm^−1^ with a 514.5 nm laser beam using a power of 0.5 mW.

#### 2.8.6. Drop Shape Analyzer

The KRUSS Drop Shape Analyzer was used along with DSA25 and ADVANCE software to analyze the surface wettability of the composite. Distilled water was used as the liquid for drop deposition.

### 2.9. Adsorption Study of Ch-GO Composite

The equilibrium methylene blue uptake capacity of the Ch-GO dip-coated PAN-EDA membrane were evaluated in batch mode using fixed amounts of adsorbent in sealed sample bottles. The adsorption isotherm of methylene blue obtained via batch mode conditions was initiated with an adsorbent dosage of 2.8 mg under varying pH levels (pH set to 3, 5, 7, 9, or 11) and varying initial methylene blue concentrations (5–50 ppm). The final volume of the methylene blue solution of each sample bottle was kept at 25 mL. A 200-ppm stock solution of methylene blue dissolved in water was prepared and the other solutions were obtained through the required levels of dilution. Absorption measurements were carried out at the maximum of absorption (λ = 664 nm) and a linear calibration curve for methylene blue absorbance at variable concentrations was obtained. The adsorption isotherm of Congo red obtained via batch mode conditions was initiated with an adsorbent dosage of 10 mg at varying levels of pH (pH set to 3, 5, 6, 7, 8, or 9) and variable initial Congo red concentrations (10–70 ppm). The final volume of the Congo red solution of each sample bottle was kept at 25 mL. A 100-ppm stock solution of Congo red dissolved in water was prepared and other solutions were obtained through the required levels of dilution. Absorption measurements were carried out at the maximum of absorption (λ = 497 nm) and a linear calibration curve for Congo red absorbance at variable concentrations was obtained. After 48 h to achieve equilibrium, the samples were separated from the solution using vacuum separation to avoid the presence of any particles and the absorbance of residual dye was measured using UV-vis spectroscopy (UV-vis 3600 spectrophotometer). The equilibrium uptake of both methylene blue and Congo red were calculated using:

(1) $$Q_{e}=\frac{C_{0}-C_{e}}{W}\times v$$

where Q_e_ (mg/g) is the weight of dye adsorbed per unit mass of adsorbent, C_0_ (mg/L) is the initial dye concentration, C_e_ (mg/L) is the residual amount of methylene blue, V (L) is the volume of the dye solution, and W (g) is the weight of the adsorbent.

### 2.10. Adsorption Isotherm Study of Ch-GO Composite

The adsorption isotherm can be described by the isotherm models developed by Langmuir, Freundlich, Dubinin-Radushkevich, etc., to conduct an accurate percentage removal of the adsorbate. The equilibrium of a homogeneous surface where the adsorbate adsorbed to a single molecular layer is described using the Langmuir isotherm model. The Freundlich isotherm model describes the multilayer coverage of molecules of the absorbate on a heterogeneous surface of an adsorbent [57]. The equations for the Langmuir model (Equation (1)) and Freundlich model (Equation (2)) are mathematically expressed as:

(2) $$\frac{C_{e}}{q_{e}}=\frac{C_{e}}{q_{m}}+\frac{1}{K_{L}\cdot q_{m}}$$

(3) $${\mathrm{logq}}_{e}=\frac{1}{n}{\mathrm{logC}}_{e}+{\mathrm{logK}}_{F}$$

where q_e_ is the equilibrium adsorption capacity (mg/g), C_e_ is the equilibrium concentration of the adsorbate solution (mg/L), q_m_ is the maximal adsorption capacity of the adsorbent (mg/g), and K_L_ is the Langmuir adsorption equilibrium constant (L/mg), while K_F_ (mg/g) and n are Freundlich constants related to the adsorption capacity and intensity of adsorption, respectively [57]. Isotherm modeling studies for methylene blue and Congo red were conducted via a plotting of the graphs in accordance with Equations (1) and (2) using the data obtained from UV-vis experiments.

## 3. Results and Discussion

### 3.1. FTIR Analysis

**Figure 7:**
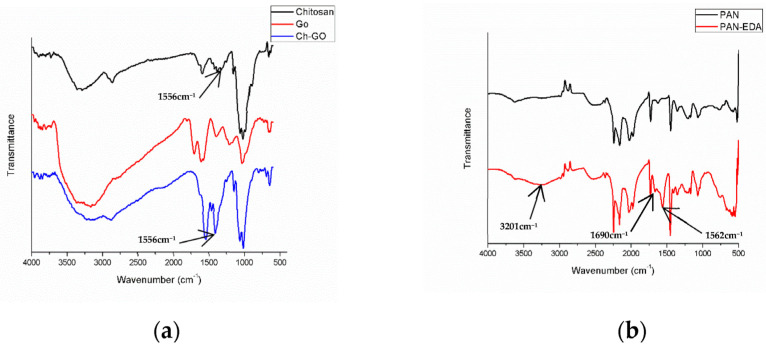
FTIR spectra of (**a**) Chitosan, Graphene Oxide, Ch-GO membrane and (**b**) PAN and PAN-EDA electrospun nanofibers.

The FTIR spectra of the synthesized chitosan depicted in Figure 7a reveals a strong band in the region 3285–3356 cm^−1^ corresponding to the N–H and O–H stretching vibrations. The small peak at 2864 cm^−1^ is indicative of the C–H stretching vibration of CH_2_ and CH_3_ groups present in the biopolymer. The peaks at 1556 and 1642 cm^−1^ are characteristic of the N–H bending and C=O stretching of the primary amine and the amide, respectively. The absorption bands located in the fingerprint region of the FTIR spectra corresponding to 1028 and 1065 cm^−1^ wavenumbers could be indicative of the C–O stretching vibrations while the peak at 1150 cm^−1^ is representative of the C–O–C asymmetric stretching vibrations in chitosan. The absorption peaks are consistent with the results reported by Laaribi et al. [57], Knidri et al. [58], Queiroz et al. [59], and Kumari and Rath [60]. The FTIR spectrum corresponding to the synthesized GO sample, i.e., Figure 7a, presents a strong peak at 3203 cm^−1^ indicative of characteristic O–H stretching vibrations. The absorption band in 1709 cm^−1^ corresponds to C=O stretching vibrations of aldehydes and ketones. The small absorptive band at 1179 cm^−1^ could be attributed to the carboxy stretching vibrations, whereas the peak corresponding to the C–O–C stretching of the epoxy groups in GO is revealed at 1036 cm^−1^. The peaks are similar to the results reported by Ciplak et al. [61], Nazri et al. [62], and Sabzevari et al. [40].

The FTIR spectra of the synthesized Ch-GO composite is also depicted in Figure 7a. There appear to be noticeable similarities between the peak locations of chitosan and the composite. The peak locations at the C–H stretching (2871–2874 cm^−1^), C–O–C stretching (1065–1148 cm^−1^), and O–H and N–H stretching bonds (3203–3249 cm^−1^) outline the stated similarities. This can be explained through the chemical reaction that occurs between the epoxy functional groups on GO and the primary amine groups in chitosan during which the primary amines are converted to secondary amines that yield similar FTIR results. However, upon close examination, the blue shift of N–H bending from 1556 cm^−1^ of chitosan to 1536 cm^−1^ of Ch-GO indicates the occurrence of epoxy amine reaction as confirmed in the study reported by Shao et al. [39].

**Figure 8:**
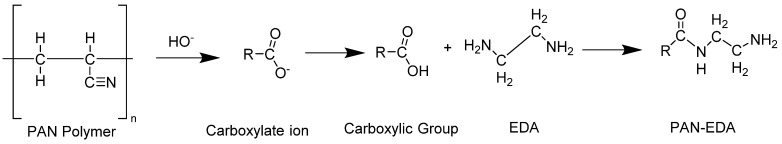
Functionalization of PAN membrane.

The FT-IR spectra of polyacrylonitrile (PAN) nanofibers and PAN functionalized with ethylenediamine (EDA) are shown in Figure 7b. The stretching and bending vibrations of the methylene groups are represented by the adsorptive peaks at 2513 and 1448 cm^−1^ in both spectra, respectively. The nitrile group in PAN is represented by the peak at 2241 cm^−1^ (stretching vibration of the CN triple bond). When PAN is reacted with Sodium Hydroxide, carboxylate ions are formed (Figure 8), which is a product of the reaction between NaOH and nitrile group (C≡N) present in PAN. When PAN is then reacted with EDA, an amide is formed, which is a result of the reaction between the amine group of EDA and carboxylate ions present in the functionalized PAN structure (Figure 8). The presence of unreacted C≡N in the PAN-EDA nanofibers causes adsorptive peaks in the range of 2241 cm^−1^. The OH bonds of hydrogen-bound water molecules could be represented by the common adsorptive band at 3629 cm^−1^. Upon close examination of the PAN and PAN-EDA spectra, the adsorptive bands at 3434, 1562, and 1690 cm^−1^, which correspond to the stretching vibration of amines, C=O stretch of the amide, and N–H bending vibration of the amide that are absent in the FTIR spectra of untreated PAN nanofibers, are present in PAN-EDA, confirming the amide formation. The results are comparable to those found in other studies by Almasion et al. [25] and Patel and Hota [56].

### 3.2. SEM Results

**Figure 9:**
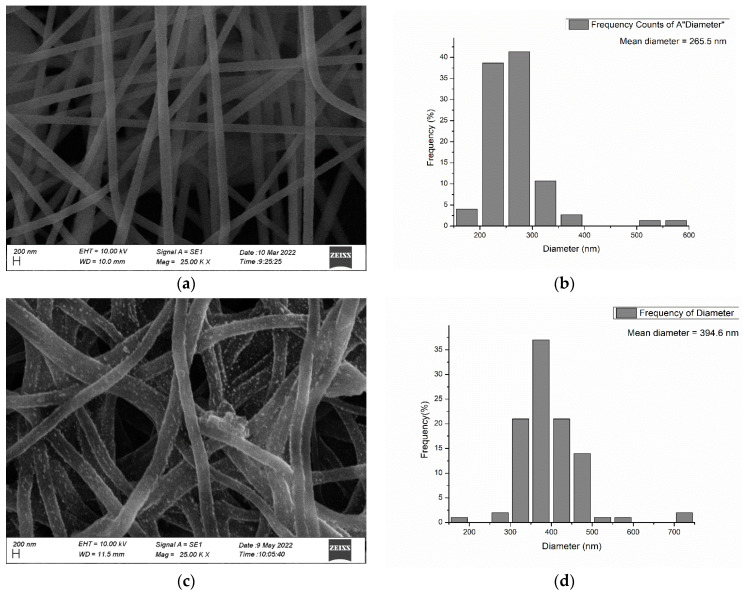
(**a**) SEM image of PAN electrospun membrane, (**b**) Histogram of PAN electrospun membrane, (**c**) SEM image of Ch-GO dip-coated PAN-EDA electrospun membrane, and (**d**) Histogram of Ch-GO dip-coated PAN-EDA electrospun membrane.

A Scanning Electron Microscope (SEM) was used to examine the surface characteristics such as the size and morphology of the PAN electrospun membrane (Figure 9a) and Ch-GO dip-coated PAN-EDA electrospun membrane (Figure 9c). Based on SEM images, the average fiber diameter of each sample was determined using the ImageJ application. Figure 9b illustrates the histogram of the PAN electrospun membrane and the average diameter of the fibers was found to be 265.5 nm. The PAN electrospun membrane consists of uniform nanofibers with smooth surfaces. The PAN electrospun membrane was functionalized with EDA and dip-coated with a Ch–GO solution. Based on Figure 9d, the average diameter of the Ch-GO dip-coated PAN-EDA electrospun fibers was 394.6 nm. The increase in diameter of the PAN nanofibers in the dip-coated nanofibers could be attributed to the swelling of PAN nanofibers following the EDA functionalization process and due to the coating of Ch-GO. The smooth surface of the PAN membrane has been converted to a rougher surface with agglomerations of graphene oxide particles (2 µm GO particle) that can be seen attached to the PAN fibers.

### 3.3. XRD and RAMAN Characterization

**Figure 10:**
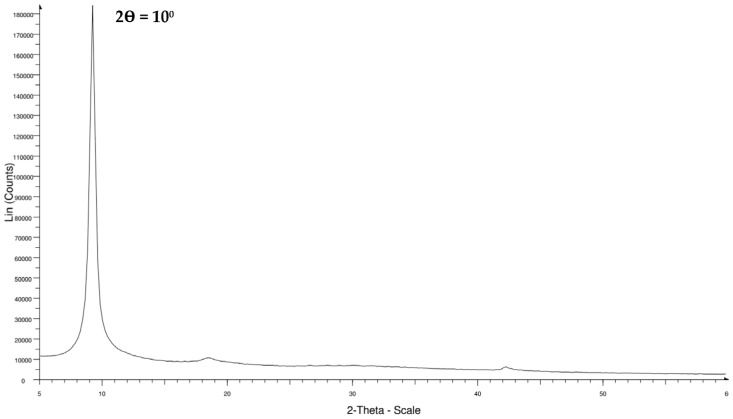
XRD spectrum of GO. (Data are from [64]).

Figure 10 depicts the XRD plot of the synthesized GO sample in which a significant peak appears to be located at 2θ = 10°, which is lower than that of the reported peak position for graphite (2θ = 26°) [63]. In conformity with Bragg’s law, it can be reasoned that the d-spacing of the GO has increased in comparison to pure graphite owing to the oxidation reaction. The intercalation of oxygen-based functional groups at the carbon basal plane, i.e., hydroxy, carbonyl, epoxy, and carboxy formed during GO synthesis, could contribute to the increment in d-spacing [64].

**Figure 11:**
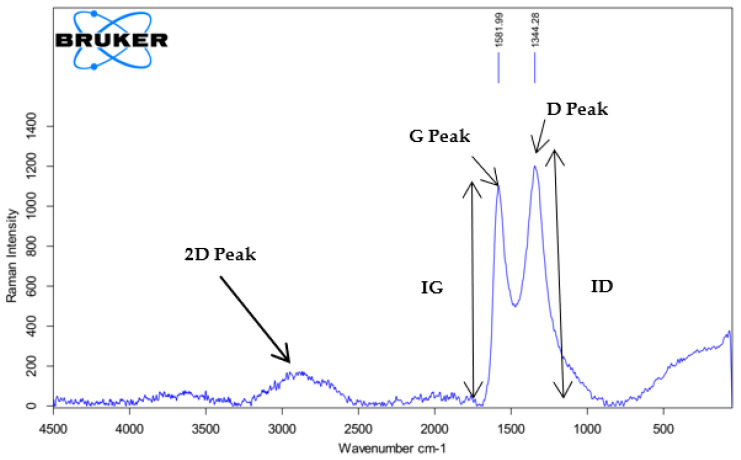
RAMAN spectrum of GO. (Data are from [63]).

The Raman spectra of the synthesized GO shown in Figure 11 displays the D, G, and 2D peaks characteristic of graphite-based materials. The graphitic G peak positioned at 1581 cm^−1^ is indicative of the primary in-plane vibrational mode. The D peak at 1344 cm^−1^ denotes the effects and defects, i.e., grain boundaries [65], vacancies, and amorphous carbon [66]. The computed ID/IG ratio amounts to 1.09, which is substantially higher than that of graphite (0.04) [67]. The ID/IG ratio of the GO synthesized in this study shows similar results to the study conducted by Jorah et al. [68]. Furthermore, the broad 2D peak located at 2700 cm^−1^ is confirmatory evidence of multilayer GO formation as reported in the previous literature [69].

### 3.4. Contact Angle Measurements

**Figure 12:**
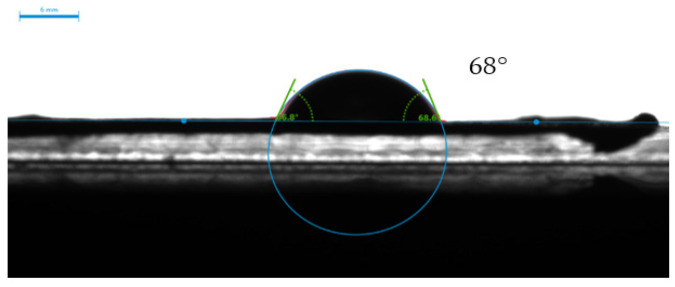
The static contact angle of water on PAN electrospun nanofiber membrane.

**Figure 13:**
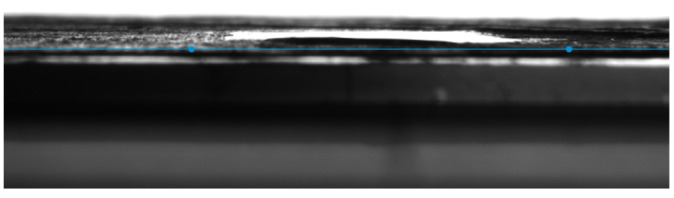
The static contact angle of water Ch-GO dip-coated PAN-EDA nanofiber membrane.

PAN is a widely used polymer to fabricate EPNs on account of its superior mechanical, chemical, and thermal properties. However, its low surface wettability hinders dye adsorption. The literature proposes surface modification as a solution to improve the hydrophilicity of polymeric nanofibers. The present study adopts two post-treatment techniques to enhance the stability of the fabricated EPN, i.e., wet chemistry and surface coating. The former refers to functionalizing PAN with EDA through a chemical reaction between the nitrile groups in PAN and amino groups in EDA while the latter refers to immersing or dip-coating the functionalized PAN membrane in a Ch-GO solution. As depicted in Figure 12, the static contact angle of the PAN electrospun membrane measured using the sessile drop method was 68° and, subsequent to surface modification, the fabricated EPN demonstrated perfect wettability (Figure 13), i.e., the static contact angle approached 0°. As reported in the literature, the hydrophilicity of an EPN will depend on the polarity, homogeneity, and layer thickness [24]. The polarity imparted by GO likely contributes toward the enhancement in hydrophilicity. It can be concluded through deductive reasoning that the improved surface wettability would contribute favorably toward the dye adsorption capacity of the membrane.

### 3.5. Adsorption Study

The absorption measurements were carried out at λ = 664 nm for methylene blue and λ = 497 nm for Congo red and linear calibration curves for methylene blue and Congo red absorbance at variable concentrations were obtained.

#### 3.5.1. Effect of Solution pH on Adsorption

**Figure 14:**
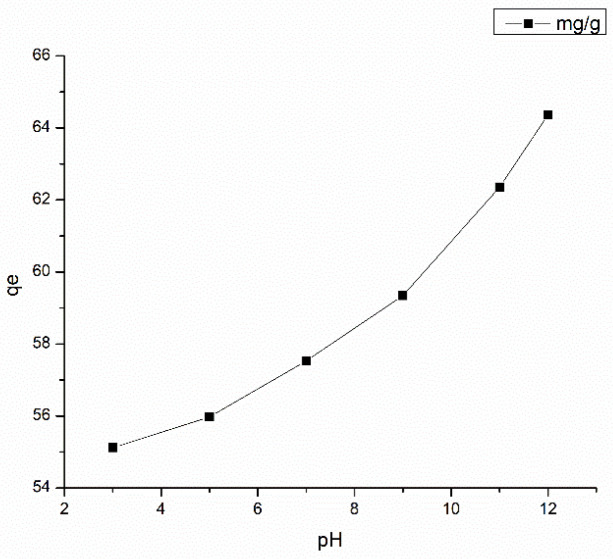
Effect of pH on methylene blue adsorption at Adsorbent dosage = 2.8 mg, Temperature = 300 K, and Contact time = 48 h.

The pH of the solution influences the ionization of GO. To determine the influence of pH on the adsorption capacity of the Ch-GO dip-coated PAN-EDA electrospun membrane toward methylene blue, a 5 ppm initial concentration of methylene blue was prepared in which the pH of the solution was varied between 3 and 10 via a dropwise addition of 0.1 M NaOH/0.1 M HCl solutions. As depicted in Figure 14, the adsorption capacity increased with rising levels of pH, which can be attributed to the increase in the surface ionization of carboxyl groups present on GO. As carboxylic acids are categorically weak acids, by nature they undergo partial dissociation in aqueous mediums yielding carboxylate and hydrogen ions. On the basis of Lei Chatelier’s principle, the addition of OH^−^ ions or a base to the solution would shift the equilibrium to the reaction side with carboxylate ions to counteract the change. Therefore, an increase in the proportion of carboxylate ions in the solution would ultimately contribute to an elevation in the electrostatic attraction force between the fabricated EPN membrane and methylene blue. Moreover, at lower pH values, the amine groups on PAN as well as in chitosan would undergo protonation generating cationic –NH_3_^+^ groups that would repel the cationic dyes in the solution yielding undesirable results. It can be concluded that a highly basic pH is favorable for the fabricated adsorbent to facilitate methylene blue removal. The results obtained with variations in pH reported in this study are consistent with the studies reported by Yang et al. [69] and Huang et al. [70].

**Figure 15:**
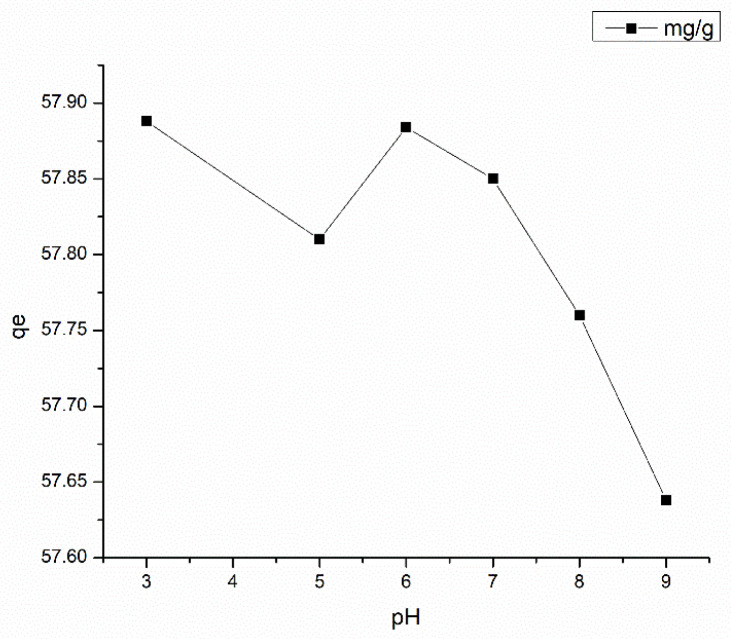
Effect of pH on Congo red adsorption at Adsorbent dosage = 2.8 mg, Temperature = 300 K, and Contact time = 48 h.

The pH of the solution regulates the protonation or the deprotonation of amino functional groups on PAN and chitosan. To study the effect of pH on the adsorption capacity of the fabricated electrospun membrane toward Congo red, a solution with an initial Congo red concentration of 20 ppm was used with an adsorbent dosage of 10 mg/25 mL. The pH level of the solution was adjusted between 3–10 using 0.1 M NaOH/0.1 M HCl solutions. As depicted in Figure 15, the adsorptive capacity of the EPN was highest at a pH value of three, fluctuates until six, and then gradually declines as the pH rises. The amino groups on PAN and chitosan are subjected to protonation at lower pH values, which enhances the electrostatic attraction between the anionic dye and the adsorbent. Conversely, as the amine group deprotonates at increasing pH, the negative charge on the functionalized surface rises. As a result of electrostatic repulsion, the proportion of total dye removal decreases sharply [71]. The results for pH variations experimentally determined in this study are consistent with the studies reported by Patel and Hota [29]. Even though the highest adsorptive capacity for Congo red is reported at a pH level of 3, there was a visible color change in the dye from red to blue at this region. As reported by Nadjia et al. [72], the maxima band in UV-vis spectroscopic studies for Congo red does not significantly shift in the pH range 6–10, which is consistent with the current study. Therefore, the optimum pH level for Congo red removal is considered to be 6.

Methylene blue shows the best adsorption capacity at higher pH values and Congo red around pH 6. The pH of the effluent after the final wash of dyeing in the textile industries is pH 7–8 [73]. Considering all these factors, the neutral pH 7 was chosen as the pH to conduct the adsorption isotherm experiments.

#### 3.5.2. Effect of Contact Time

**Figure 16:**
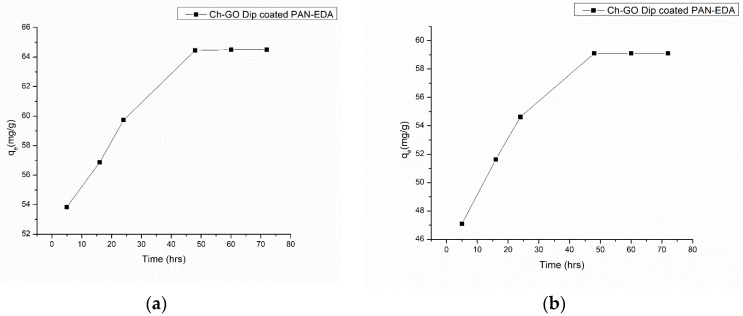
Effect of contact time on (**a**) methylene blue and (**b**) Congo red adsorption at Adsorbent dosage = 2.8 mg, Temperature = 300 K, and pH = 7.

A sufficient contact time is required to reach the equilibrium of the dye adsorption. Additionally, as per the results provided in Figure 16a,b, both methylene blue and Congo red dyes required a duration of 48 h to achieve the equilibrium. The adsorption capacity of methylene blue was 64.45 mg/g, which is higher than that of the Congo red, 59.1 mg/g at the equilibrium. The effect of temperature on the adsorptive capacity of methylene blue and Congo red dyes onto the Ch-GO dip-coated PAN-EDA membrane were investigated by varying the temperature, but no significant change in the equilibrium concentration was observed.

### 3.6. Adsorption Isotherm

**Figure 17:**
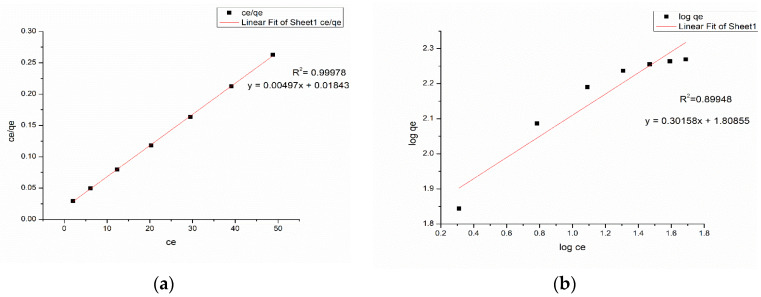
(**a**) Langmuir model (**b**) Freundlich for methylene blue at Adsorbent dosage = 2.8 mg, pH = 7, Temperature = 300 K, and Contact time = 48 h.

**Figure 18:**
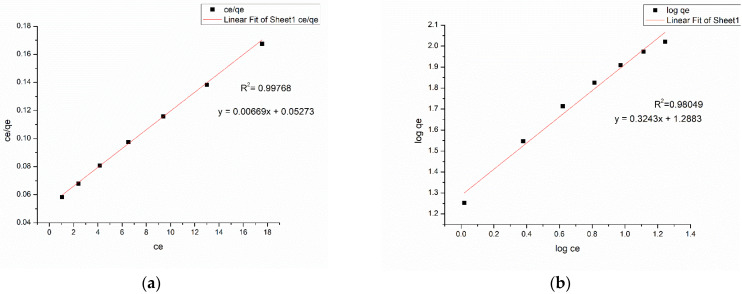
(**a**) Langmuir model and (**b**) Freundlich for Congo red at Adsorbent dosage = 2.8 mg, pH = 7, Temperature = 300 K, and Contact time = 48 h.

The isotherms for the Langmuir and Freundlich models for methylene blue and Congo red are displayed in Figure 17 and Figure 18. The equilibrium parameters corresponding to the two models deduced from the two isotherm fittings are listed in Table 1. Table 2 and Table 3 detail a comparative study of the maximum adsorption capacities (q_max_) of adsorbents reported in the literature targeting methylene blue and Congo red removal. 

**Figure 19:**
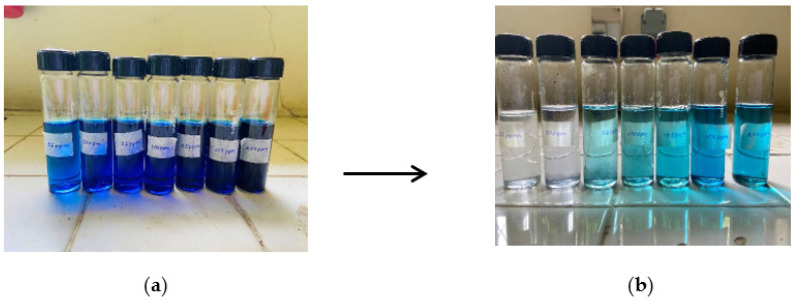
Methylene blue solutions (**a**) before and (**b**) after adsorption. The concentrations were 25 ppm, 50 ppm, 75 ppm, 100 ppm, 125 ppm, 150 ppm, and 200 ppm (from left to right).

The higher correlation coefficient (R^2^ = 0.99978) of the Langmuir model for methylene blue dye implies that the adsorption of methyelene blue onto the nanocomposite membrane is best suited with a Langmuir isotherm model, which is indicative of a monolayer, homogenous adsorption. Figure 19a and Figure 19b illustrate the methylene blue solutions before and after dye adsorption. The experimentally determined q_max_ of the Ch-GO dip-coated PAN-EDA electrospun membrane amounts to 201.20 mg/g, which is significantly higher than that of raw chitosan (11 mg/g) [69] and slightly less than GO powder (243 mg/g) [36]. The slight reduction in the adsorption capacity of the fabricated nanocomposite membrane toward methylene blue in comparison to the GO powder can be attributed to the decrease in the available epoxy functional sites owing to the epoxy-amino reaction. Nonetheless, the nanocomposite membrane exhibits superior adsorption capacity to other carbon-based nanomaterials, i.e., graphene [74] and carbon nanotubes (CNTs) [75], which is due to the enhancement in the adsorption capacity imparted by the oxygen-containing functional groups on GO distributed over a large surface area. Moreover, it can also be observed that the q_max_ of the fabricated adsorbent surpasses the other chitosan-graphene oxide composites reported in the literature [31,37,76]. As reported in research studies, organic dye removal from aqueous systems using pristine, blended, or modified EPNs occurs via pi-pi stacking, hydrogen binding, and pore-filling [33,42,51,77,78,79]. Due to the presence of a nitrile group, PAN possesses the ability to attract positively charged dyes through dipole interaction [80]. As reported by Haider et al. [81], PAN displays a maximum adsorptive capacity of 42.67 mg/g toward methylene blue, which can be used to explain the increase in the adsorptive capacity of the Ch-GO dip-coated PAN-EDA electrospun membrane toward methylene blue in comparison to the other chitosan-graphene oxide-based composites reported in the literature. In the present study, the PAN electrospun membrane is modified via the addition of EDA during which the nitrile group undergoes a chemical reaction (Figure 8). As discussed under the FTIR analysis, the unreacted nitrile groups may participate in hydrogen bond formation with methylene blue. Moreover, the reaction between NaOH and PAN causes the formation of an intermediate carboxylate ion that is negatively charged. The unreacted carboxylate ions present on the PAN surface may also contribute to the formation of electrostatic attraction forces with the cationic dye molecule. The q_max_ of the fabricated EPN toward methylene blue also exceeds that of other PAN-functionalized electrospun membranes reported in the literature [79]. Through deductive reasoning, it can be concluded that the nanocomposite membrane discussed in the present study is an effective adsorbent for methylene blue.

**Figure 20:**
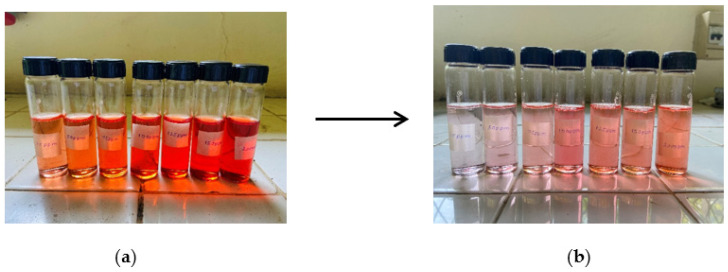
Congo red solutions (**a**) before and (**b**) after adsorption. The concentrations were 25 ppm, 50 ppm, 75 ppm, 100 ppm, 125 ppm, 150 ppm, and 200 ppm (from left to right).

The adsorption of Congo red onto the Ch-GO dip-coated PAN-EDA electrospun membrane also follows a Langmuir isotherm model owing to the higher correlation coefficient (R^2^= 0.99904) and the q_max_ was experimentally determined to be 151.74 mg/g. Figure 20a and Figure 20b illustrate the congo red solutions before and after dye adsorption. The electrostatic attraction forces between the protonated amine groups and the negatively charged Congo red governs the dye removal capacity of the nanocomposite membrane. According to the data provided in Table 3, it can be determined that the q_max_ of the fabricated EPN for Congo red removal exceeds that of the PAN-EDA electrospun membrane reported by the studies of Patel and Hota [29]. This can be attributed to the presence of the chitosan top coating that provides additional protonated amino sites for anionic dye removal. Chitosan is a promising and cost-effective adsorbent for dye removal renowned for its biodegradability and non-toxicity; however, its raw form does not possess a higher surface area, which limits its adsorption capacity [81]. In the present study, a chitosan-graphene oxide layer is coated over a large surface area, which, therefore, provides the potential to increase its adsorption capacity. The fabricated EPN displays a superior adsorption capacity to other adsorbents reported in the literature as listed in Table 3. Therefore, it can be concluded that the present study provides an effective solution for Congo red removal from aqueous systems. 

**Table 2:** Comparison of the adsorption capabilities of several adsorbents for methylene blue dye in the literature.

Absorbent	Adsorption Capacity (mg/g)	Sources
CNTs	46.2	[82]
Graphene	153.9	[83]
β-cyclodextrin/MGO	93.97	[84]
Graphene/SrAl_2_O_3_.Bi^3+^	42.92	[85]
GO-cyclodextrin-chitosan-Fe_3_O_4_	84	[33]
GO-chitosan-Fe_3_O_4_	95	[75]
GO-Fe_3_O_4_	167	[76]
GO/Co_3_O_4_ nanocomposite	40	[86]
Magnetic reduced graphene oxide loaded hydrogels	119	[87]
Raw Chitosan	11	[69]
Graphene Oxide powder	243	[70]
PAN Nanofibers	42.67	[78]
Oxime—grafted PAN	102.15	[79]
Ch-GO dip-coated PAN-EDA Electrospun Nanofiber Membrane	201.2	Present work

**Table 3:** Comparison of the adsorption capabilities of several adsorbents for Congo red dye in the literature.

Absorbent	Adsorption Capacity (mg/g)	Sources
A/Zn/PAN Nanofibers	25.64	[88]
4-VP grafted PET fibers	17.3	[89]
Functionalized PVC/Graphene-polyaniline fibers	40.0	[90]
ZnO/SnO_2_ porous nanofibers	90.8	[91]
SiO_2_—AlOOH (Bohemite) core/sheath fibres	24.3	[92]
Chitosan hydrobeads	92.59	[93]
Commercial Activated carbon	66.67	[94]
Palladium nanoparticles loaded on AC	76.9	[95]
PAN-EDA functionalized membrane	130	[29]
Ch-GO dip-coated PAN-EDA Electrospun Nanofiber Membrane	151.745	Present work

### 3.7. Reusability Study

Conducting regeneration studies is crucial for the adsorbent to be reusable and to reduce the cost incurred in wastewater treatment. The dye-adsorbed Ch-GO dip-coated PAN-EDA membrane was treated with a 0.01 M NaOH solution and then continuously stirred for two hours at 150 rpm to allow the adsorbed dyes to separate from the composite in order to study the regeneration capacity of the manufactured adsorbent. Afterward, the adsorbent was repeatedly cleaned using deionized water before being dried in an oven for one hour at 60 °C. The adsorptive removal of dyes during the following cycle reused the nanocomposite membrane.

**Figure 21:**
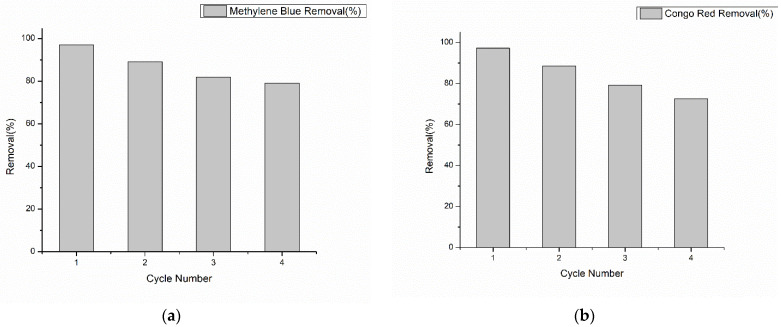
(**a**) Methylene blue and (**b**) Congo red dye removal percentage as a function of cycle number.

The investigation of the methylene blue and Congo red dye adsorption using the regenerated Ch-GO dip-coated PAN-EDA membrane occurred during four successive cycles and the outcomes for both dyes are shown in Figure 21a and Figure 21b. It was demonstrated that, up to the fourth cycle, more than 79.1% of methylene blue and 72.5% of Congo red were removed. As a result, it is shown that the Ch-GO dip-coated PAN-EDA membrane is an effective and highly reusable adsorbent for the decontamination of methylene blue and Congo red dyes.

## 4. Conclusions

A Ch-GO dip-coated electrospun nanofiber membrane was successfully fabricated to facilitate methylene blue and Congo red removal. FT-IR spectroscopy was used to confirm the occurrence of cross-linking between the epoxy-amino groups on the Ch-GO coating and verify the amide formation via the reaction between nitrile groups in PAN and EDA in the presence of NaOH. The SEM images revealed that the average diameter of PAN increased from 265.5 nm to 394.6 nm after surface modification, which can be attributed to the swelling following the EDA functionalization process as well due to the coated Ch-GO layer. In terms of the nanofiber morphology, it was also revealed that the uniform surface of PAN was transformed to a relatively rougher exterior following surface modification. Contact angle measurements were recorded using the sessile drop method confirmed that the modified PAN electrospun membrane displayed perfect wettability owing to the successful coating of the Ch-GO tier. The effect of the pH, temperature, and contact time on methylene blue and Congo red removal was studied and the following conclusions were deduced: the adsorption process for methylene blue is more favorable in a basic medium due to the ionization of carboxyl groups on GO, while an acidic medium was favorable for Congo red removal due to the protonation of amine groups; the temperature does not significantly influence the adsorption process for both dyes; and the two reactions achieved equilibrium after a significant time interval. The maximum adsorptive capacities of methylene blue and Congo red onto the fabricated EPN membranes were determined to be 201.20 mg/g and 151.74 mg/g, respectively, which were following a Langmuir isotherm model for a monolayer and homogenous adsorption. The novel Ch-GO dip-coated electrospun nanofiber membrane fabricated to facilitate methylene blue and Congo red removal has proven to be effective and superior to many other adsorbents reported in the literature.

## 5. Future Perspectives

Advancements in the field of nanotechnology offer a promising future for modified polyacrylonitrile electrospun membranes in the field of wastewater treatment applications. This is primarily attributable to the versatile design capabilities accessible via electrospinning. The design or the morphology of the nanofibrous structure can be altered to suit the requirement of the user through the precise manipulation of electrospinning parameters, i.e., applied voltage, polymer concentration, tip-to-collector distance, feed rate, temperature, and humidity. The designed adsorbent outlined in the present study offers prospects for future developments. Future research can be focused on improving the mechanical strength, reusability, regeneration, and durability of electrospun membranes designed for wastewater applications. The mechanical strength of the membrane is generally correlated with its thickness, which can be improved via long-term electrospinning and using alternative techniques such as needle electrospinning or multi-needle electrospinning. Another strategy is to stack several electrospun mats on one another, but its adherence can be weak. Therefore, considerable research should be focused to improve the mechanical properties of EPNs. One such suggestion would be to design and develop electrospun membranes with a non-woven base. The non-woven bottom layer may contribute to increased strength and durability while facilitating microfiltration. The adsorption capacity of the nanostructure toward methylene blue and Congo red outlined in the present study could be improved by incorporating porous PAN nanofibers in place of regular PAN nanofibers to enhance the surface area available for adsorption. As an added functionality, the fabricated structure could be modified to facilitate the removal of heavy metal ions from wastewater streams. Even though many advancements in the topic of upscaling potential or mass production of electrospun polymer membranes are reported in the literature, the potential for further improvements as its usage in a practical context remains indeterminant. In general, modified electrospun nanofibers are expected to be a trending research topic with promising future prospects.

## Figures and Tables

**Table 1:** Results of Langmuir and Freundlich isotherms.

	Langmuir Isotherm			Freundlich Isotherm		
Dye	q_max_ (mg/g)	K_L_ (L/mg)	R^2^	K_F_	n	R^2^
Methylene blue	201.2072	0.269669	0.99978	1.0397	3.2927	0.89948
Congo red	151.745	0.000348	0.99904	1.1218	3.0835	0.98049

## Data Availability

Data available on request.
